# Functional analysis of the mRNA profile of neutrophil gelatinase-associated lipocalin overexpression in esophageal squamous cell carcinoma using multiple bioinformatic tools

**DOI:** 10.3892/mmr.2014.2465

**Published:** 2014-08-07

**Authors:** BING-LI WU, CHUN-QUAN LI, ZE-PENG DU, FEI ZHOU, JIAN-JUN XIE, LIE-WEI LUO, JIAN-YI WU, PI-XIAN ZHANG, LI-YAN XU, EN-MIN LI

**Affiliations:** 1 Department of Biochemistry and Molecular Biology, Shantou University Medical College, Shantou, Guangdong 515041, P.R. China; 2 College of Bioinformatics Science and Technology, Harbin Medical University, Harbin, Heilongjiang 150081, P.R. China; 3 Department of Pathology, Shantou Central Hospital, Affiliated Shantou Hospital of Sun Yat-sen University, Shantou, Guangdong 515041, P.R. China; 4 Department of Biochemistry and Molecular Biology, Guangdong Pharmaceutical University, Guangzhou Higher Education Mega Center, Guangzhou, Guangdong 510000, P.R. China; 5 Institute of Oncologic Pathology, Shantou University Medical College, Shantou, Guangdong 515041, P.R. China

**Keywords:** neutrophil gelatinase-lipocalin, esophageal squamous cell carcinoma, mRNA profile, bioinformatic analysis

## Abstract

Neutrophil gelatinase-associated lipocalin (NGAL) is a member of the lipocalin superfamily; dysregulated expression of *NGAL* has been observed in several benign and malignant diseases. In the present study, differentially expressed genes, in comparison with those of control cells, in the mRNA expression profile of EC109 esophageal squamous cell carcinoma (ESCC) cells following *NGAL* overexpression were analyzed by multiple bioinformatic tools for a comprehensive understanding. A total of 29 gene ontology (GO) terms associated with immune function, chromatin structure and gene transcription were identified among the differentially expressed genes (DEGs) in *NGAL* overexpressing cells. In addition to the detected GO categories, the results from the functional annotation chart revealed that the differentially expressed genes were also associated with 101 functional annotation category terms. A total of 59 subpathways associated locally with the differentially expressed genes were identified by subpathway analysis, a markedly greater total that detected by traditional pathway enrichment analysis only. Promoter analysis indicated that the potential transcription factors *Snail*, *deltaEF1*, *Mycn*, *Arnt*, *MNB1A*, *PBF*, *E74A*, *Ubx*, *SPI1* and *GATA2* were unique to the downregulated DEG promoters, while *bZIP910*, *ZNF42* and *SOX9* were unique for the upregulated DEG promoters. In conclusion, the understanding of the role of *NGAL* overexpression in ESCC has been improved through the present bioinformatic analysis.

## Introduction

Neutrophil gelatinase-associated lipocalin (NGAL), also termed lipocalin2, is a member of the lipocalin superfamily, which includes >20 members (1). NGAL is secreted extracellularly and forms a heterodimer with matrix metalloproteinase-9 (MMP-9) through disulfide bonds protecting against degradation (2). NGAL tightly binds to the bacterial siderophore, possibly serving as a potent bacteriostatic agent by sequestering iron as well as regulating innate immunity and inflammation (3). Overexpression of *NGAL* has also been observed in various types of human cancer, including breast, colorectal, pancreatic, ovarian, gastric, thyroid, ovarian, bladder and kidney cancer (4). Previous studies have shown that *NGAL* is upregulated in esophageal squamous cell carcinoma (ESCC) and is an independent prognostic factor; this upregulation was significantly correlated with cell differentiation and tumor invasion (5,6).

However, controversial results have been observed regarding the functional role of NGAL in various types of cancer cell. For example, NGAL was able to facilitate gastrointestinal mucosal regeneration by promoting cell motility and invasion and to reduce E-cadherin mediated cell-cell adhesion in colon cancer (7). NGAL was demonstrated to be highly expressed in human thyroid carcinomas, and NGAL knockdown inhibited cancer cell growth in soft agar and the formation of tumors in nude mice (8). Conversely, in pancreatic cancer cells, NGAL reduced adhesion/invasion partly through suppressing focal adhesion kinase activation and inhibited angiogenesis partly by blocking vascular endothelial growth factor production (9).

In the present study, to examine the biological role of NGAL in ESCC, *NGAL* was overexpressed in the EC109 ESCC cell line. An mRNA microarray was performed using the Agilent whole genome oligo microarray to identify differentially expressed genes (DEGs) in *NGAL* overexpressing cells compared with control cells (10). Multiple bioinformatics analyses were performed on these DEGs in order to gain a comprehensive understanding of the role of *NGAL* overexpression in ESCC.

## Materials and methods

### Differentially expressed genes

The raw data were analyzed using normalization and log transformation (10). Differentially expressed genes were identified using a two-fold change threshold.

### Gene ontology (GO) enrichment and functional annotation

The Database for Annotation, Visualization and Integrated Discovery bioinformatics tool (DAVID; http://david.abcc.ncifcrf.gov/) was applied for GO enrichment, using category classes including Biological process, Cellular component and Molecular function. GO is one of the most useful methods for functional annotation and classification of genes. In addition, DAVID bioinformatics provides a functional annotation chart to identify over-represented biological terms from a particular gene list (11). Thus far, the functional annotation chart provides >40 category enrichments, including GO terms, sequence features, disease associations, protein functional domains, protein-protein interactions, pathways, homology, gene functional summaries and literature. The enriched terms from the functional annotation chart with P<0.05 were visualized by the Enrichment Map plugin for the Cytoscape network visualization software (12).

### Kyoto Encyclopedia of Genes and Genomes (KEGG) pathway and subpathway analysis

The bioconductor SubpathwayMiner package was applied to the DEG-enriched KEGG pathways identified (12). In addition to traditional entire pathway enrichment, SubpathwayMiner is able to detect subpathways, local regions of entire pathways, which aids in gaining more detailed information regarding the relevant genes in localized areas of a specific pathway (13). SubpathwayMiner extracts multiple subpathways from an entire KEGG pathway by the k-clique method. The distance between any two nodes (a node indicates a gene in the pathway) in a subpathway is not larger than k; k was set as 4 in the present study.

### Promoter sequence patterns and potential transcription factor analysis

The 2,000-bp promoter sequences of the 20 genes exhibiting the greatest down- and upregulation, respectively, were retrieved from the UCSC genome database (http://genome.ucsc.edu/). The sequence patterns over-represented or under-represented in these two promoter sequence sets were analyzed by the POCO program (http://ekhidna.biocenter.helsinki.fi/poxo/poco/poco). POCO identifies motifs that are over-represented in one dataset compared with a background set, but under-represented in another dataset compared with the same background set. For the parameters in the present study, the background organism was set as homo_sapiens_clean and the longest pattern length was set as 8. Subsequently, significant sequence patterns were screened in the JASPAR transcription factor database (http://jaspar.binf.ku.dk) to identify recognized transcription factors (similarity index >0.70) (14).

## Results

### GO enrichment and functional annotation

**Figure 1:**
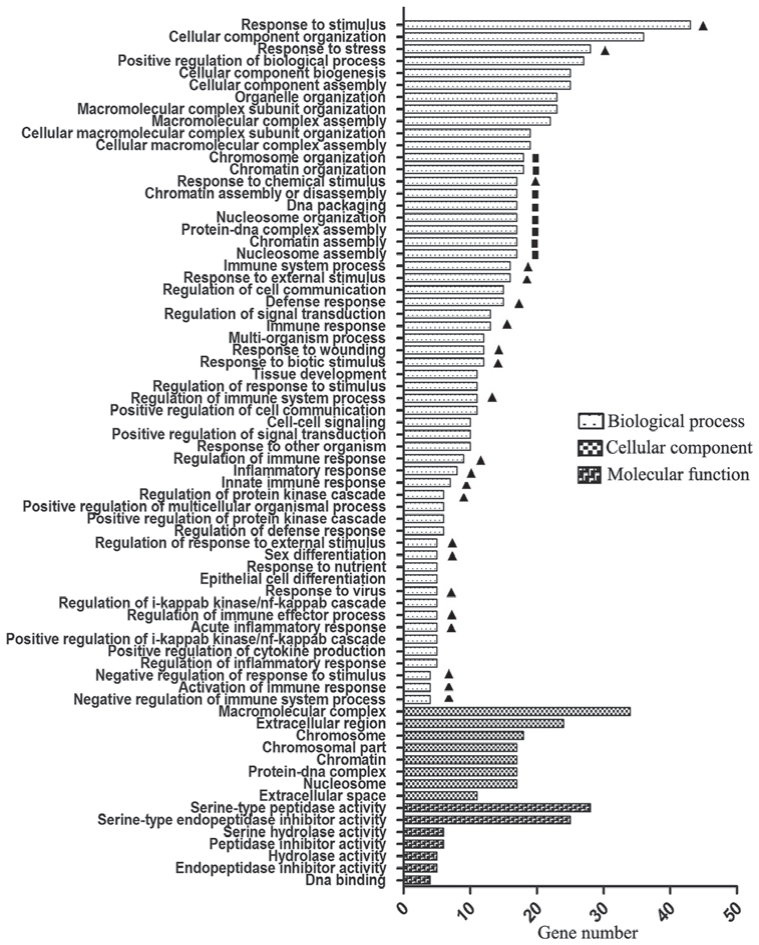
Gene ontology enrichment of the differentially expressed genes from the mRNA expression profile of neutrophil gelatinase-associated lipocalin overexpressing EC109 esophageal squamous cell carcinoma cells, when compared with control cells. The terms from the Biological process, Cellular component and Molecular function categories are signified by different patterned bar graphs. Immune-associated terms and histone-associated terms are indicated by triangles and squares, respectively.

A total of >200 DEGs in the *NGAL* overexpressing cells were obtained using a two-fold change as the threshold, including 167 upregulated genes and 96 downregulated genes (Table I). To determine the functional classification of the various gene clusters, GO annotation was conducted using DAVID, which constructs statistically significant functional profiles from a set of genes. A total of 75, 8 and 7 significantly enriched GO terms were identified for these DEGs in the Biological process, Cellular component and Molecular function categories, respectively (P<0.05; Fig. 1). Notably, two predominant Biological process term groups were identified. One group comprised 21 immune-associated terms, including response to stress, defense response and regulation of immune response. The other group consisted of 8 terms regarding chromatin structure and gene transcription, including nucleosome assembly, chromatin assembly and protein-DNA complex assembly. These 8 terms contained the same 17 DEGs: *HIST1H2AC*, *HIST2H2AA3*, *HIST1H2BB*, *HIST1H2BC*, *HIST1H1E*, *HIST1H2BD*, *HIST1H1C*, *HIST1H2BE*, *HIST1H2AG*, *HIST1H2BF*, *HIST1H2BG*, *HIST1H2AD*, *HIST1H2BH*, *HIST1H2BO*, *HIST1H2BM*, *H2BFS*, *HIST1H2BK*, *HIST1H2BL*, *HIST1H2BI*, *HIST2H2AC*, *HIST1H3D* and *HIST3H2BB*. The most significant function in the Molecular function category was DNA-binding; in addition to 17 histone-associated genes, this contained *ZMAT1*, *IFIH1*, *LMO2*, *TFCP2L1*, *SOX2*, *TP63*, *DACH1*, *FOXN4*, *TAF11* and *OASL*. In the Cellular component category, a total of 23 genes associated with the extracellular region were identified: *SECTM1*, *RBP4*, *A2M*, *C3*, *CFB*, *PLBD1*, *LGALS8*, *SPINK5*, *APOL3*, *CGREF1*, *SLC1A3*, *ISG15*, *SAA1*, *SERPINA5*, *AGT*, *C1RL*, *KLK10*, *IGFL2*, *AGRN*, *SEPP1*, *AREG*, *CASP1* and *DEFB1*.

**Figure 2:**
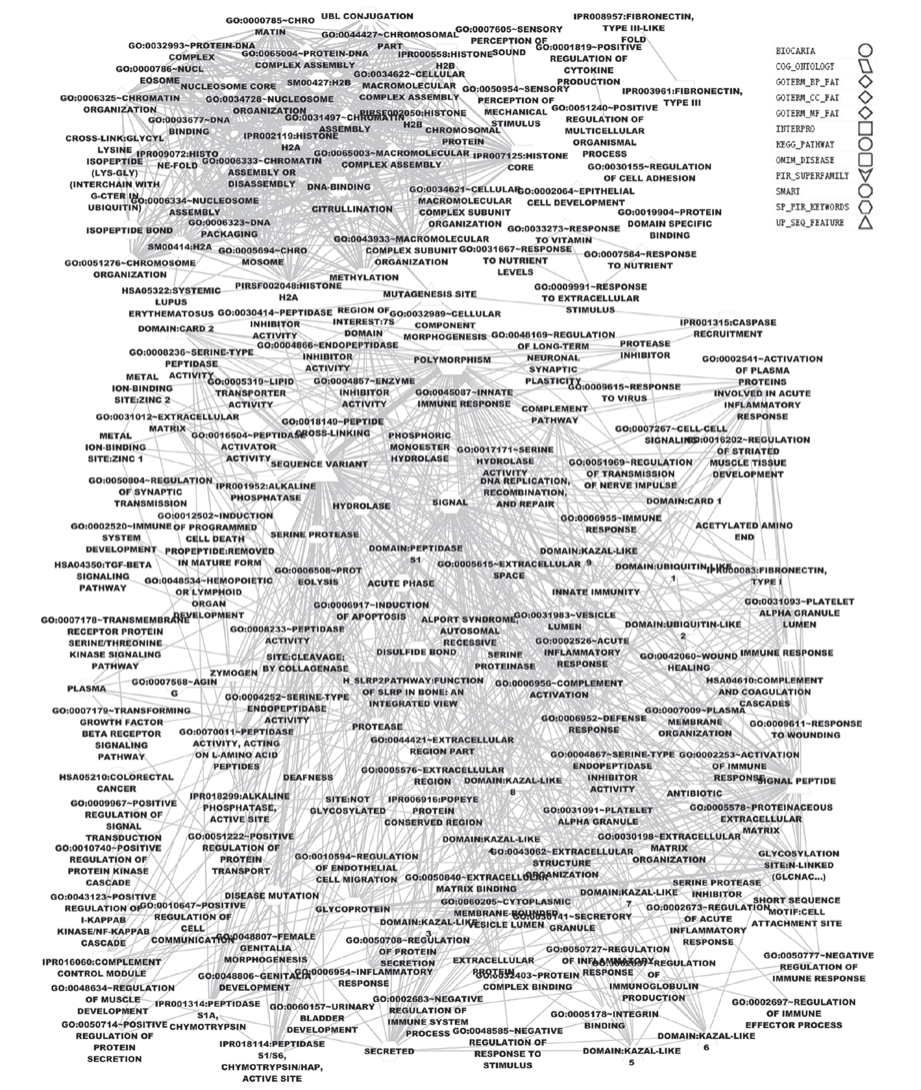
Functional categories of the differentially expressed genes were visualized using the Enrichment map plugin of the Cytoscape network visualization software. A significant functional term is signified by one node with size indicating the enrichment significance P-value. Edges indicate gene overlap between nodes and thickness indicates the size of the overlap. Nodes from the same functional category are presented as the same shape.

The DEGs were also clustered using the Functional annotation chart in DAVID and the enrichment was visualized by the Enrichment Map plugin for the Cytoscape software. In Fig. 2, a node signifies one functional category and node size corresponds to the number of enriched genes. The color depth corresponds to the significance (P-value) of the terms. Nodes from the same functional category are presented as the same shape. Edges between nodes were depicted when overlapping genes existed between these two nodes. The widths of the lines indicate the number of overlapping genes between the functional groups, which are bigger and the wider with greater numbers. In the 180 total Functional annotation chart enrichments identified, in addition to 101 terms from the three GO categories, 78 terms from the following annotation categories were included: 14 from INTERPRO, 2 from SMART, 30 from SP_PIR_KEYWORDS, 24 from UP_SEQ_FEATURE, 1 from COG_ONTOLOGY, 2 from PIR_SUPERFAMILY, 1 from OMIM_DISEASE and 4 from KEGG_PATHWAY. These results provided a wider overview of the biological impact of *NGAL* overexpression in ESCC than traditional GO enrichment. Five DEGs were identified in the *Homo sapiens* (hsa)04350:TGF-beta signaling pathway term, including *SMAD9*, *ACVRL1*, *TGFBR1*, *DCN* and *TGFB1*. The autosomal recessive Alport syndrome is a genetic condition characterized by kidney disease, hearing loss and eye abnormalities. The majority of affected individuals experience progressive loss of kidney function, usually resulting in end-stage kidney disease. This disease was detected in the OMIM_DISEASE category containing two risk genes, *COL4A4* and *COL4A3* (15). In the SP_PIR_KEYWORDS category, 67 genes were enriched when using the Signal term. In addition, 35 genes were observed to be enriched using the Secreted term in SP_PIR_KEYWORDS. Four genes (*C3*, *SAA1*, *CFB* and *FN1*) were enriched in the acute phase term in SP_PIR_KEYWORDS. The ubl conjugation term in SP_PIR_KEYWORDS contained 27 genes; in addition to 15 histone-associated genes, this also included another 12 genes: *TSHZ2*, *SOX2*, *TP63*, *FOS*, *H2BFS*, *INSIG1*, *COL4A3*, *SGK1*, *DDX58*, *PJA1*, *MIB2* and *ADD3*. The only enrichment term in COG_ONTOLOGY was DNA replication, recombination and repair, which contained four genes (*DDX58*, *IFIT1*, *IFIH1* and *DDX60*).

### Pathway and subpathway enrichment

The DEGs were mapped to KEGG pathways to identify the cell signaling pathways influenced by the downstream effectors of *NGAL*. The DEGs were enriched in only four pathways (Table II).

**Figure 3:**
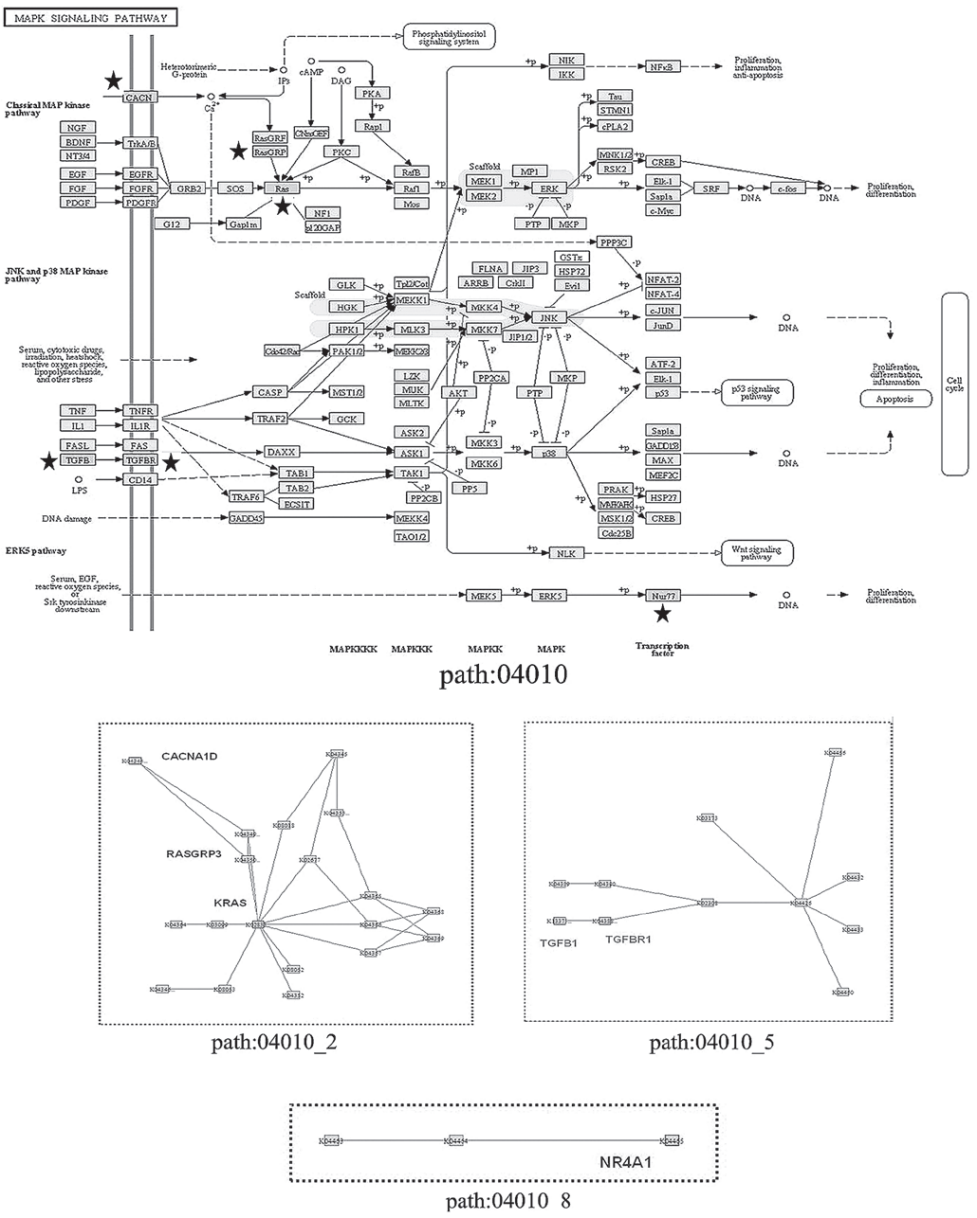
Differentially expressed gene-enriched subpathway in the MAPK signaling pathway (path:04010). The six differentially expressed genes are indicated by a black star in the entire origin pathway. The subpathway structures of path:04010_2, path:04010_5 and path:04010_8 generated by Subpathway package are also shown. MAPK, mitogen-activated protein kinase.

The local area of an entire pathway was able to be defined by multiple subpathways using the node distance k, which aids in understanding how the indicated genes affect the pathway locally. The DEGs were found to be significantly enriched in 60 subpathways corresponding to 27 entire pathways using the SubpathwayMiner package (Table III). Of note, the mitogen-activated protein kinase (MAPK) signaling pathway (has: 04010) was not detected by the entire pathway enrichment, but was found to be significant in the subpathway analysis, with three subpathways derived from three local areas of this signaling pathway (Fig. 3). The subpathway path:04010_2 contained three DEGs: *RASGRP3*, *KRAS* and *CACNA1D*; path:04010_5 contained *TGFB1* and *TGFBR1*, while path:04010_8 only contained *NR4A1*. Another pathway detected using this analysis was the TGF-beta signaling pathway (has:04350), which was not identified by entire KEGG pathway enrichment, but four subpathways were detected. Path:04350_6 contained *DCN*, *TGFB1* and *TGFBR1*. Path:04350_4 and path:04350_7 contained *DCN* and *TGFB1*, while path:04350_1 and path:04350_8 contained *SMAD9* and *TGFBR1*.

### Promoter sequence patterns and potential transcription factors in upregulated and downregulated genes

**Figure 4:**
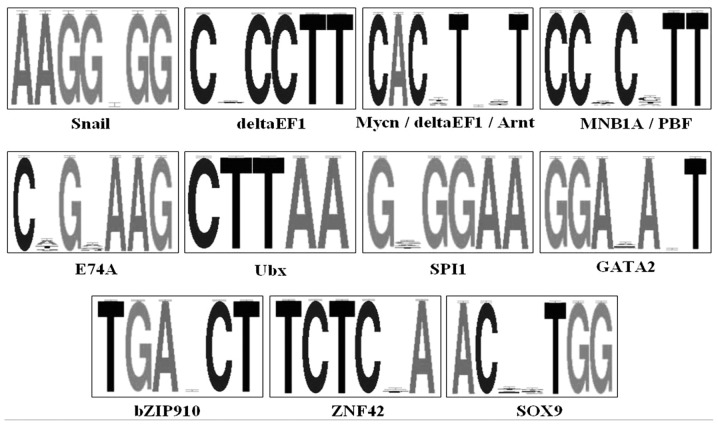
Sequence pattern logos of the predicted transcription factors. To identify recognized transcription factors, the distinguishing significant patterns were screened using the JASPAR transcription factor database. The potential transcription factors regulating downregulated genes are presented in the upper two panels, while the transcription factors regulating downregulated genes are listed in the third panel.

The spatial distribution and abundance of promoter *cis*-elements affects gene expression. The co-expression of upregulated and downregulated genes in *NGAL* overpressing ECO109 cells was considered to be regulated by specific transcription factors at the transcriptional level. POCO is a software program that is able to identify over-represented and under-represented regulatory patterns among promoter sequence sets of upregulated and downregulated genes. In the present study, a total of 52 significant sequence patterns were identified to be over-represented in the downregulated genes but comparatively under-represented in the upregulated genes, of which the top 20 patterns are presented in Table IV. Conversely, 75 patterns were observed to be over-represented in the upregulated genes and simultaneously under-represented in the downregulated genes; the top 20 patterns are shown in Table V. The identified patterns were 5–8 bp long, containing the four known nucleotides, A, C, G and T, while the rest of the places in a pattern, marked as N, may be any of these (which are variable). Subsequently, all significant patterns were screened with the JASPAR transcription factor database to identify potential transcription factors. A total of 11 patterns corresponding to 14 unique transcription factors were detected (Fig. 4). Of these potential transcription factors, *Snail*, *deltaEF1*, *Mycn*, *Arnt*, *MNB1A*, *PBF*, *E74A*, *Ubx*, *SPI1* and *GATA2* were unique for the downregulated DEG promoters, while *bZIP910*, *ZNF42* and *SOX9* were unique for the upregulated DEG promoters. These results indicated that these transcription factors may be associated with specific transcriptional regulation in the downregulated and upregulated DEGs. Although a number of sequence patterns did not correspond to known transcription factors, the possibility and importance in the regulation of DEGs subsequent to *NGAL* overexpression was not discounted.

## Discussion

ESCC has one of the highest mortality rates of malignant tumors worldwide, particularly in Asia, with an overall five-year survival rate <20% (16). NGAL has been shown to be an important mediator of invasion and metastasis in ESCC (5,6,10). However, for a improved understanding of the role of NGAL in ESCC, a comprehensive analysis of the mRNA profile of *NGAL* overexpression ESCC cells was conducted in the present study, using multiple bioinformatic analyses. A total of 267 DEGs were observed in the *NGAL* overexpressing cells compared with control cells, using a two-fold change as the threshold. To understand the function of these DEGs, the DEGs were analyzed by GO enrichment using DAVID bioinformatics. Several GO terms associated with known NGAL functions were detected. For example, 21 immune-associated terms were identified, including response to stress, defense response and regulation of immune response. In the response to stimulus (GO:0050896) term, >43 genes were enriched. For example, one of the enriched genes, *RAD9*, protects against genomic instability by activating DNA damage checkpoint and DNA damage repair pathways (17). Another enriched gene, *DEFB1*, is constitutively expressed in epithelial tissues, but may be upregulated upon receiving inflammatory or microbial stimuli (18).

Recent studies have observed that NGAL is involved in the antibacterial iron-depletion strategy of the innate immune system. NGAL binds catecholate-type siderophores, such as enterobactin synthesized by *E. coli,* to arrest *E. coli* growth through inhibiting the iron-uptake ability (19). Several studies found NGAL to be critical in the antimicrobial molecular response in infections, including *Salmonella* (20,21), *Chlamydia* (22) and *Mycobacterium tuberculosis* (23). The GO enrichment analysis in the present study suggested that in addition to NGAL itself, NGAL downstream effectors exert a marked impact on cell immune function and in response to other stimuli, including stress and defense responses.

Of note, 17 histone-associated proteins were upregulated in response to *NGAL* overexpression. The association between NGAL and histone-associated proteins had not been reported previously, to the best of our knowledge. Therefore, investigating how NGAL influences chromatin structure and gene transcription was of interest. The results of the present study provided novel information regarding the role of NGAL in gene transcriptional regulation through chromatin organization and nucleosome assembly.

The functional annotation chart provided a markedly wider overview of the biological impact of *NGAL* overexpression in ESCC than traditional GO enrichment. The chart reported that five DEGs were found using the hsa04350:TGF-beta signaling pathway term, which were not identified by the KEGG pathway enrichment analysis. Alport syndrome, which contained *COL4A4* and *COL4A3*, was the only enriched term from the OMIM_DISEASE category listed in the chart. Urine and plasma NGAL have been revealed to be novel biomarkers for diagnosis and outcome prediction in renal dysfunction conditions, including acute kidney injury, chronic kidney disease and renal ischemia-reperfusion injury (24–26). The correlation between kidney disease and NGAL interaction with downstream effectors was marked. A total of 67 genes were enriched in the SP_PIR_KEYWORDS signal term and 33 of these genes were contained in the Secreted term.

NGAL is a secreted protein, which forms a complex with MMP-9 to prevent its autodegradation, which is critical for extracellular matrix remodeling (2). Extracellular NGAL has been suggested to cause the secretion of other proteins, such as FN1, which regulate the acute inflammatory response, cell-matrix adhesion and the defense response (27). Four genes, *C3*, *SAA1*, *CFB* and *FN1*, were enriched in the SP_PIR_KEYWORDS acute phase term. Of note, all four genes are defined as positive acute phase proteins, which are considered to exert the following general functions: Opsonization and trapping of microorganisms and associated microbial products; binding cellular remnants, such as nuclear fractions; scavenging free hemoglobin and radicals; and modulating the immune response of the host (28).

Although an entire pathway may not be identified to be statically significant, alterations in local gene expression levels may affect the local pathway significantly, which results in a marked impact on the biological outcome. Subpathway analysis is a powerful method to detect genes in the local area of the KEGG pathway. Li *et al* (29) constructed a drug-metabolic subpathway network and found the local region of the tyrosine metabolic pathway to be closely associated with the development of lung cancer. A total of 60 subpathways corresponding to 27 entire pathways were found in the present study. Several subpathway-derived entire pathways were identified using this method. For example, the MAPK signaling pathway and the TGF-beta signaling pathway were detected. These results suggested that although certain DEGs did not significantly affect an entire pathway, they did perturb the pathway locally. Other proteins in these pathways were not differentially expressed at the mRNA level, but this may exclude processes such as modification and complex formation, undergone by the DEGs.

DEGs were classified into upregulated and downregulated genes as determined by the respective expression levels. How these two group genes were co-regulated by distinguishing sequence patterns and transcription factors was notable. The POCO software program identifies over- and under-represented regulatory patterns among the promoter sequence sets of upregulated and downregulated genes. Not all DEGs are considered to be modified at the transcriptional level; the DEGS may have been differentially expressed due to differences in mRNA stability. Thus, in the present study, the 20 genes exhibiting the greatest up- or downregulation in *NGAL* overexpressing ESCC cells were analyzed by POCO. Hundreds of significant sequence patterns and dozens of transcription factors were found to be over- and under-represented in the downregulation gene set and the upregulation gene set, respectively. This suggested that the change in signal transduction following *NGAL* overexpression resulted in specific transcription factors and/or certain sequence patterns exerting critical regulatory roles, to achieve co-regulation of the significantly down- or upregulated genes at the transcriptional level.

A number of these potential transcription factors have previously been reported to be associated with cancer invasion or metastasis. *Snail* and *ZEB1* (*deltaEF1*) are predominantly involved in the repression of E-cadherin expression, resulting in epithelial to mesenchymal transition, which has been implicated as the critical event initiating cancer invasion and metastasis (30,31). Overexpression of *Snail* was shown to correlate positively with lymphovascular invasion and was associated with poorer overall survival in ESCC patients (32). Nuclear expression of *ZEB1* was observed in >33% ESCC tumor cells, while *ZEB1* was not detected in the normal adult esophageal epithelia (33). *PBF* was hypothesized to induce the translocation of PTTG to the cell nucleus, where it induces tumorigenesis via a number of different mechanisms (33). *PBF* is upregulated by estrogen and mediates estrogen-stimulated cell invasion in breast cancer cells (34). SPI1 co-operates with MYC regulating the transcription of *microRNA-29b*, which is important in the neutrophil differentiation of acute promyelocytic leukaemia cells (35). Notably, NGAL was first identified as a protein stored in specific granules of human neutrophils (36). A potential SPI1 binding site was identified in the promoter region of the *NGAL* gene by computer analysis (37). These results indicated that SPI1 may be the key molecule in biological functions mediated by NGAL. SOX9, a high-mobility group box transcription factor, is required for development, differentiation and lineage commitment. Cytoplasmic SOX9 may serve as a valuable prognostic marker in invasive ductal carcinoma and metastatic breast cancer. The significant correlation identified between SOX9 and breast tumor cell proliferation implies that SOX9 directly contributes to the poor clinical outcomes associated with invasive breast cancer (38). These results indicated that these transcription factors may be involved in the invasion or metastasis mediated by NGAL. Although numerous sequence patterns were not matched to known transcription factors, the specific base composition suggested that these patterns may be crucial in transcriptional regulation. These results indicated that these sequence patterns and transcription factors may respond to particular transcriptional regulation in downregulated and upregulated DEGs.

In conclusion, in the present study, a comprehensive understanding of the role of NGAL in ESCC following *NGAL* overexpression was obtained by multiple bioinformatic analyses, particularly through analyzing subpathway and sequence patterns for co-expression, which provided more information than traditional methods. These analytical methods may be used to search for novel functional genes and pathways associated with the relevant genes identified from high-throughput data.

## Figures and Tables

**Table I:** Differentially expressed genes in neutrophil gelatinase-associated lipocalin overexpressing EC109 esophageal squamous cell carcinoma cells, compared with control cells.

A, Upregulated genes	

Gene symbol	Fold change
*LCN2*	75.450
*BC034319*	12.960
*CGREF1*	8.069
*SLC1A3*	7.525
*CLGN*	5.594
*SECTM1*	5.505
*POPDC3*	5.469
*FNDC6*	5.215
*FXYD3*	5.102
*KLK10*	4.260
*CHST2*	4.142
*UBD*	4.122
*LGALS8*	4.084
*DEFB1*	3.971
*PLEKHA4*	3.910
*CSAG1*	3.817
*LGALS8*	3.719
*SPINK5*	3.665
*C1orf38*	3.520
*RPSAP10*	3.464
*PCDHB5*	3.436
*DUSP26*	3.416
*DDX58*	3.376
*LMO2*	3.368
*CFB*	3.352
*FOXN4*	3.336
*BTN3A3*	3.279
*BF514799*	3.245
*AGRN*	3.240
*KIAA0657*	3.177
*GLRX*	3.074
*NAALAD2*	3.067
*LOC389772*	3.046
*DACH1*	3.044
*TP73L*	3.026
*TCEAL2*	3.016
*PADI1*	2.991
*DYSF*	2.987
*METTL7A*	2.977
*SERPINA5*	2.956
*POF1B*	2.934
*SAMD9L*	2.913
*MGC16075*	2.908
*RASGEF1A*	2.904
*RBP4*	2.895
*IPO13*	2.894
*TTTY22*	2.888
*HIST1H1C*	2.781
*C15orf59*	2.777
*HERC6*	2.774
*PLK2*	2.761
*CSAG3A*	2.752
*IFI27*	2.734
*DOCK11*	2.729
*BLOC1S1*	2.723
*SLC22A4*	2.704
*IFIH1*	2.696
*C3*	2.647
*ABCA1*	2.627
*OASL*	2.608
*ZMAT1*	2.607
*UNC5B*	2.572
*FBP1*	2.560
*HIST1H2BK*	2.560
*CAMSAP1L1*	2.559
*PFTK1*	2.553
*HIST1H1E*	2.550
*IFIT2*	2.544
*KYNU*	2.530
*RADIL*	2.495
*KYNU*	2.492
*IFIT1*	2.490
*PPAPDC3*	2.484
*HIST1H2BC*	2.480
*ABCA1*	2.480
*CA8*	2.468
*AREG*	2.467
*ADD3*	2.455
*AF264621*	2.451
*TDRD9*	2.445
*LOC375010*	2.433
*BX115350*	2.433
*GRAMD1C*	2.430
*SEPP1*	2.427
*ALPPL2*	2.416
*SMIM1*	2.408
*LOC391566*	2.406
*SAA1*	2.390
*AKR1C3*	2.379
*HIST2H2AA*	2.370
*IFI44*	2.366
*REEP6*	2.361
*PREX1*	2.357
*S100A3*	2.355
*SOX2*	2.348
*C9orf9*	2.341
*H2BFS*	2.340
*RIMS4*	2.330
*HIST1H2BH*	2.316
*HIST1H2BF*	2.310
*PSMB9*	2.307
*LOC375010*	2.290
*HIST1H2BE*	2.286
*FLJ20035*	2.285
*CSTA*	2.283
*HIST1H2BB*	2.281
*DIO3OS*	2.270
*HIST1H2BM*	2.268
*NID67*	2.260
*FAM31C*	2.257
*HKDC1*	2.253
*ZMAT1*	2.246
*HIST1H2BL*	2.237
*PLSCR4*	2.233
*HIST1H2BD*	2.223
*HIST1H2BI*	2.217
*RAD9B*	2.215
*C9orf9*	2.211
*BTN3A1*	2.211
*HIST1H3D*	2.210
*HERC5*	2.205
*TSGA2*	2.204
*BC021677*	2.204
*HIST1H2BG*	2.203
*GPNMB*	2.200
*PLBD1*	2.187
*PAG1*	2.177
*G1P2*	2.175
*TPO*	2.172
*HIST1H2BN*	2.163
*A2M*	2.161
*CACNA1D*	2.156
*AGT*	2.148
*NANOS1*	2.147
*SLC5A10*	2.138
*CASP1*	2.138
*FAM26F*	2.126
*PSD3*	2.116
*KARCA1*	2.112
*CR596233*	2.099
*HIST1H2BO*	2.094
*PLEKHA4*	2.094
*APOL3*	2.084
*BC043357*	2.076
*CXorf48*	2.076
*HIST2H2AC*	2.076
*AF074986*	2.074
*HIST1H2AD*	2.074
*MGC16075*	2.068
*IGFL2*	2.064
*C1RL*	2.060
*TAF11*	2.048
*PHEX*	2.048
*MGC45474*	2.047
*ABCG2*	2.046
*ADPRHL1*	2.043
*HIST1H2AG*	2.043
*OSTbeta*	2.042
*POPDC2*	2.042
*RHBDL4*	2.040
*GPR126*	2.037
*TFCP2L1*	2.036
*PJA1*	2.035
*BBS5*	2.035
*HRASLS*	2.035
*C18orf56*	2.028
*PLCG2*	2.017
*MIB2*	2.014
*KRAS*	2.004

B, Downregulated genes	

Gene symbol	Fold change

*CHST6*	0.0931
*ZNF521*	0.110
*IFI16*	0.155
*IFI16*	0.173
*DIAPH2*	0.173
*SEMA5A*	0.204
*CENTA2*	0.205
*CENTA2*	0.212
*CPM*	0.216
*KAL1*	0.228
*DCN*	0.237
*CXXC4*	0.250
*FOXQ1*	0.252
*FOS*	0.268
*COL4A3*	0.287
*TMEPAI*	0.294
*GUCY1A2*	0.300
*CLU*	0.312
*LEPREL2*	0.316
*LYPDC1*	0.323
*RASGRP3*	0.323
*TMEM46*	0.342
*OLFML2A*	0.344
*RGS5*	0.359
*PLAT*	0.360
*MASK*	0.363
*TMEPAI*	0.367
*SCARA3*	0.368
*DISC1*	0.369
*SPFH2*	0.370
*FOXP1*	0.370
*FOXP1*	0.381
*FSTL4*	0.383
*NPTX1*	0.383
*CDK6*	0.387
*FAM211B*	0.391
*GPR56*	0.394
*OR51B4*	0.400
*SERPINE2*	0.400
*ATP9B*	0.400
*FZD10*	0.402
*NPTX1*	0.404
*CR597240*	0.410
*NFKBIZ*	0.410
*CRLF1*	0.426
*GPR56*	0.426
*ALPL*	0.428
*SQLE*	0.429
*NALP1*	0.432
*TGFBR1*	0.434
*MGC4294*	0.434
*COL4A4*	0.435
*KLK1*	0.435
*FAM101B*	0.437
*SLC7A13*	0.439
*PGCP*	0.441
*LOC155060*	0.451
*MCTP2*	0.452
*SGK*	0.452
*FN1*	0.455
*EGR1*	0.455
*ANKH*	0.456
*SDC2*	0.457
*RDH10*	0.458
*XYLT1*	0.458
*TGFBI*	0.458
*FRY*	0.459
*BAPX1*	0.460
*TGFB1*	0.460
*HBG1*	0.462
*EDIL3*	0.462
*SLC38A5*	0.464
*TRPM4*	0.468
*PMP22*	0.468
*FLJ21986*	0.470
*NR4A1*	0.470
*CPM*	0.471
*ADAMTS5*	0.475
*HBG1*	0.476
*XAGE2*	0.478
*TSHZ2*	0.479
*INSIG1*	0.479
*RGS22*	0.484
*KCNQ1*	0.485
*HMGCS1*	0.490
*MYO1A*	0.490
*HMGCS1*	0.490
*LOC284542*	0.494
*PTPRB*	0.496

**Table II:** Enriched Kyoto Encyclopedia of Genes and Genomes DEG pathways.

Pathway ID	Pathway	annMolecule Ratio^a^	P-value
05322	Systemic lupus erythematosus	20/268	0.0000
04610	Complement and coagulation cascades	5/268	0.0016
05210	Colorectal cancer	4/268	0.0075
00790	Folate biosynthesis	2/268	0.0077

a annMolecule ratio is how many genes are enriched in a pathway. The first number indicates the number of annotated DEGs in the pathway. The second number signifies the total number of molecules in the pathway.

DEG, differentially expressed gene.

**Table III:** Enriched Kyoto Encyclopedia of Genes and Genomes subpathways of differentially expressed genes in neutrophil gelatinase-associated lipocalin overexpressing EC109 esophageal squamous cell carcinoma cells.

Entire pathway ID	Entire pathway	Subpathway ID	P-value
Path:04960	Aldosterone-regulated sodium reabsorption	path:04960_3	0.0161
		path:04960_2	0.0462
Path:05146	Amoebiasis	path:05146_8	0.0124
Path:04662	B cell receptor signaling pathway	path:04662_9	0.0002
		path:04662_4	0.0005
Path:05142	Chagas disease	path:05142_7	0.0483
Path:05220	Chronic myeloid leukemia	path:05220_5	0.0015
Path:05210	Colorectal cancer	path:05210_7	0.0077
Path:04610	Complement and coagulation cascades	path:04610_7	0.0008
		path:04610_1	0.0043
		path:04610_6	0.0043
		path:04610_4	0.0375
		path:04610_2	0.0403
		path:04610_3	0.0403
		path:04610_5	0.0432
Path:04060	Cytokine-cytokine receptor interaction	path:04060_22	0.0015
		path:04060_44	0.0244
Path:04623	Cytosolic DNA-sensing pathway	path:04623_1	0.0364
Path:04512	ECM-receptor interaction	path:04512_12	0.0064
		path:04512_21	0.0364
		path:04512_23	0.0364
		path:04512_24	0.0483
Path:04012	ErbB signaling pathway	path:04012_9	0.0168
Path:00790	Folate biosynthesis	path:00790_1	0.0022
		path:00790_4	0.0022
		path:00790_5	0.0030
		path:00790_2	0.0040
Path:05160	Hepatitis C	path:05160_8	0.0364
Path:04730	Long-term depression	path:04730_5	0.0271
Path:04010	MAPK signaling pathway	path:04010_5	0.0161
		path:04010_8	0.0364
		path:04010_2	0.0393
Path:05218	Melanoma	path:05218_6	0.0322
		path:05218_3	0.0492
Path:04621	NOD-like receptor signaling pathway	path:04621_4	0.0009
		path:04621_7	0.0364
		path:04621_6	0.0483
Path:05223	Non-small cell lung cancer	path:05223_4	0.0432
Path:05212	Pancreatic cancer	path:05212_9	0.0040
Path:05200	Pathways in cancer	path:05200_25	0.0040
		path:05200_18	0.0224
		path:05200_3	0.0248
Path:04145	Phagosome	path:04145_2	0.0483
Path:04622	RIG-I-like receptor signaling pathway	path:04622_1	0.0027
		path:04622_7	0.0202
		path:04622_3	0.0296
Path:05150	Staphylococcus aureus infection	path:05150_1	0.0224
		path:05150_2	0.0224
		path:05150_7	0.0348
		path:05150_4	0.0483
Path:00140	Steroid hormone biosynthesis	path:00140_14	0.0483
Path:04660	T cell receptor signaling pathway	path:04660_6	0.0064
		path:04660_7	0.0107
Path:04350	TGF-beta signaling pathway	path:04350_6	0.0035
		path:04350_4	0.0142
		path:04350_7	0.0296
		path:04350_1	0.0403
		path:04350_8	0.0462
Path:04270	Vascular smooth muscle contraction	path:04270_13	0.0483

ECM, extracellular matrix; MAPK, mitogen-activated protein kinase; NOD, nucleotide-binding oligomerization domain; RIG, retinoic acid-inducible gene; TGF, transforming growth factor.

**Table IV:** Sequence patterns over-represented in the downregulated genes, but under-represented in the upregulated genes.

Pattern	OCC1 (#PRO/#TOT)	OCC2 (#PRO/#TOT)	F-score	P-value
TGNGGNAA	42 (19/20)	14 (11/18)	3803.53	3.33E-04
CTNNGCTT	36 (19/20)	12 (10/18)	3370.77	9.24E-04
CACNNNTT	116 (20/20)	58 (18/18)	3160.89	1.52E-03
TTAANG	107 (20/20)	42 (13/18)	3118.93	1.67E-03
CTTCNCNC	43 (19/20)	13 (9/18)	3107.02	1.72E-03
AAGGNG	140 (20/20)	65 (18/18)	3000.42	2.21E-03
CCNCCTT	54 (20/20)	19 (10/18)	2823.35	3.36E-03
TTAANGNA	48 (19/20)	14 (9/18)	2771.02	3.80E-03
CTNNCNTA	71 (20/20)	35 (15/18)	2702.61	4.47E-03
AANGNGNG	106 (20/20)	54 (17/18)	2665.29	4.88E-03
GACANNT	84 (20/20)	40 (15/18)	2637.85	5.21E-03
AANNNGNG	372 (20/20)	265 (18/18)	2629.26	5.32E-03
GNNAAGA	146 (20/20)	84 (17/18)	2585.59	5.90E-03
CANNCNTT	104 (20/20)	50 (16/18)	2579.94	5.97E-03
TNTCCNC	149 (20/20)	86 (18/18)	2575.13	6.04E-03
GTGGNNAG	43 (19/20)	15 (10/18)	2562.63	6.22E-03
GAAAGNC	35 (18/20)	13 (10/18)	2530.94	6.71E-03
CACNCNTT	31 (19/20)	10 (8/18)	2452.32	8.08E-03
ACANNTNC	108 (20/20)	56 (15/18)	2447.07	8.18E-03
GNANNANG	402 (20/20)	277 (18/18)	2380.87	9.57E-03

OCC, total number of patterns within the corresponding cluster sequences; OCC1, downregulated DEG promoter sequence set; OCC2, upregulated DEG promoter sequence set; PRO, total number of sequences with the pattern in the corresponding cluster; TOT, total number of sequences in the corresponding cluster; F-score, analysis of variance between the two input clusters and the background sequence set. DEG, differentially expressed gene.

**Table V:** Sequence patterns over-represented in the upregulated genes, but under-represented in the downregulated genes.

Pattern	OCC1 (#PRO/#TOT)	OCC2 (#PRO/#TOT)	F-Score	P-value
CTCNA	276 (20/20)	355 (18/18)	5070.50	9.19E-04
ACNNCANT	55 (19/20)	97 (18/18)	4985.61	1.05E-03
CTCA	331 (20/20)	476 (18/18)	4740.31	1.54E-03
TNNAGTCC	10 (10/20)	31 (18/18)	4712.78	1.61E-03
CAANCT	56 (19/20)	109 (18/18)	4363.70	2.77E-03
TNCTNAC	60 (19/20)	103 (18/18)	4182.29	3.68E-03
TCTCA	80 (20/20)	124 (18/18)	4112.35	4.10E-03
TNNTNGAG	66 (20/20)	111 (18/18)	4083.41	4.29E-03
GGNNTCAA	15 (12/20)	42 (18/18)	3998.49	4.90E-03
CTCANT	79 (19/20)	130 (18/18)	3985.14	5.01E-03
TGAGNNA	103 (20/20)	158 (18/18)	3861.85	6.07E-03
CTCAA	66 (20/20)	115 (18/18)	3716.14	7.63E-03
ANNGGNGT	55 (19/20)	99 (18/18)	3684.44	8.02E-03
TTNGAG	78 (20/20)	116 (18/18)	3519.73	1.04E-02
TGTNANC	64 (18/20)	122 (18/18)	3507.74	1.06E-02
ANACC	213 (20/20)	278 (18/18)	3458.47	1.14E-02
TGGNNTC	77 (19/20)	128 (18/18)	3384.73	1.28E-02
CCAANCT	11 (8/20)	33 (18/18)	3379.39	1.29E-02
TTGANNC	53 (19/20)	93 (18/18)	3372.46	1.31E-02
CCNANNNT	285 (20/20)	337 (18/18)	3362.13	1.33E-02

OCC, total number of patterns within the sequences of the corresponding cluster; OCC1, downregulated DEG promoter sequence set; OCC2, upregulated DEG promoter sequence set; PRO, total number of sequences with the pattern in the corresponding cluster; TOT, total number of sequences in the corresponding cluster; F-score, result of the analysis of variance between the two input clusters and the background sequence set. DEG, differentially expressed gene.
